# Interfering with transmission

**DOI:** 10.7554/eLife.37552

**Published:** 2018-05-24

**Authors:** Andreas Wack

**Affiliations:** Francis Crick InstituteLondonUnited Kingdom

**Keywords:** Influenza virus, interferons, transmission, respiratory tract, Mouse, Virus

## Abstract

The IFNλ family of interferons controls the spread of viruses in the upper respiratory tract and transmission between mice.

**Related research article** Klinkhammer J, Schnepf D, Ye L, Schwaderlapp M, Gad HH, Hartmann R, Garcin D, Mahlakõiv T, Staeheli P. 2018. IFN-λ prevents influenza virus spread from the upper airways to the lungs and limits virus transmission. *eLife* **7**:e33354. doi: 10.7554/eLife.33354

The evolutionary arms race between viruses and their vertebrate hosts is biased by the different rates at which each can adapt. Slowly evolving vertebrate genomes cannot keep up with the rapid mutations employed by viral genomes to avoid the host’s defenses. One strategy that vertebrates use to counter this apparent disadvantage involves a group of proteins called interferons that infected cells produce when they sense a virus. Named for their ability to ‘interfere’ with influenza virus replication (Isaacs and Lindenmann, 1957), interferons switch on a battery of hundreds of genes, many of which encode antiviral proteins (Mostafavi et al., 2016).

Interferons can be classed into different families, each of which interacts with a different receptor. The most widely studied family is the IFNαβ family, which interacts with the IFNαβ receptor (McNab et al., 2015). The IFNλ family of interferons, which interact with the IFNλ receptor, were discovered more recently (Wack et al., 2015). Now, in eLife, Peter Staeheli of the University of Freiburg and co-workers – including Jonas Klinkhammer and Daniel Schnepf as joint first authors – report a unique role for IFNλ in virus transmission (Klinkhammer et al., 2018).

IFNαβ and IFNλ activate largely the same genes, but their receptors are not found on all cell types. Therefore, the distribution of IFNαβ and IFNλ receptors could be behind the different biological effects of each interferon family. Epithelia are thought to express both receptors, and therefore the two interferon families should both have the same effects in the epithelial cells that line the surfaces of organs, including the airways. A number of findings support this. First, the influenza virus triggers the airway epithelia of mice lacking one type of receptor to transcribe practically the same sets of genes as those transcribed in the epithelia of normal mice: however, epithelia that lack both interferon receptors have large 'holes' in their transcriptional profile and allow the virus to replicate extensively (Crotta et al., 2013). Second, mice that are deficient in one type of receptor experience a mild increase in their risk of developing disease when infected, whereas mice that lack both types are far more severely afflicted (Mordstein et al., 2008).

To show that IFNλ has unique roles in epithelial cells Klinkhammer et al. – who are based at Freiburg, Aarhus University and the University of Geneva – infected mice with a strain of influenza virus in a small volume of fluid. This mimics the ways that infections spread between humans better than the larger volumes of fluid used in most studies. Using this technique, infection was initially limited to the epithelia lining the nasopharyngeal cavities.

IFNλ receptor-deficient mice infected in this way had a far greater number of virus particles in their upper airways than mice that were deficient in the IFNαβ receptor. This suggests that nasopharyngeal epithelia may be more responsive as a whole to IFNλ than to IFNαβ, or that some cells in these epithelia express only the IFNλ receptor. However, IFNαβ receptors become increasingly important when mice are infected with larger droplets, which brings the virus further down into the lungs, or during an ongoing infection if the virus succeeds in spreading further down along the airways into the lung.

The efficiency with which viruses are transmitted from infected to uninfected individuals makes the difference between minor outbreaks (such as sporadic cases of H5N1) and pandemics (such as the H1N1 pandemic of 2009). A lack of a genetically versatile animal model had made it difficult to study how the genome of the host affects transmission frequency, but this changed when it was shown that H3N2 influenza viruses transmit efficiently between mice (Edenborough et al., 2012).

Using this model Klinkhammer et al. show that IFNλ determines transmission efficiency by controlling the number of virus particles at the uppermost end of the respiratory tract: the higher the number of viruses in the nasopharyngeal space, the higher the likelihood that they would be shed out of the nose and thus be able to infect a new host. In line with its role further down the respiratory tract, IFNαβ has much less influence on how well the virus spreads between individuals.

The results also have clinical relevance. Klinkhammer et al. demonstrate here that IFNλ acts more strongly than IFNαβ and has longer-lasting effects on virus transmission, while others have shown that it also produces comparably minor inflammatory side effects (Davidson et al., 2016; Galani et al., 2017). Treatment with an IFNλ spray may therefore benefit patients with influenza, as well as anyone they encounter during their illness.

There had been previous indications that IFNλ has a unique role in controlling infections. Last year it was reported that IFNλ appeared to be the first interferon, and sometimes the only interferon, required to control the influenza virus early in infections and when using low doses of the virus (Galani et al., 2017). There also seems to be a parallel situation in the small intestine where IFNλ, and not IFNαβ, determines the outcome of some viral infections (Mahlakõiv et al., 2015; Pott et al., 2011; Figure 1). Furthermore, at both sites, some epithelial cells appear to respond only to IFNλ. What are these cells? Are there more sites in the body where this response pattern is found? Do these cells express a unique pattern of receptors, or is their response determined by other mechanisms? Like all important studies, the work presented by Klinkhammer et al. answers some questions while also raising many others.

**Figure 1.**
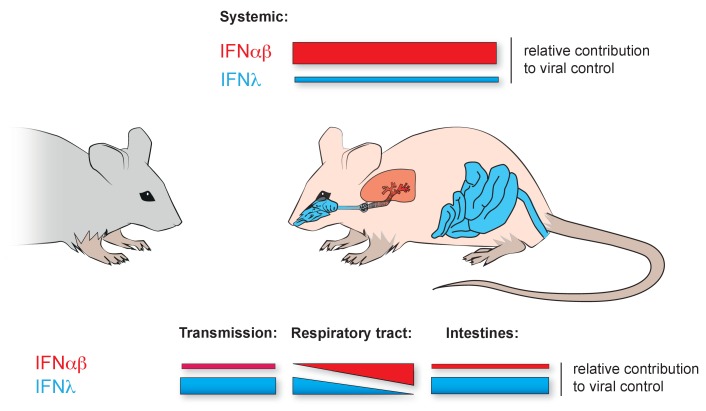
Local dominance of different interferon families in viral control. Although the IFNαβ (red) and IFNλ (blue) families of interferons activate largely similar sets of genes in response to a viral infection, their importance in fighting off infections varies in different parts of the body. The color of the organs of the mouse indicates which interferon family dominates there. The relative heights of the color-coded bars in the figure represent the extent to which each interferon family is responsible for fighting off viruses in different parts of the mouse. Top bars: In systemic infections of the mouse organism, the IFNαβ family of interferons contributes more to the control of viruses than the IFNλ family. Bottom, left: Klinkhammer et al. show that IFNλ is important for controlling the transmission of the influenza virus from infected mice to new hosts. Bottom, middle: IFNλ is also the interferon family that predominantly controls the spread of viruses in the uppermost part of the respiratory tract. However, further down the respiratory tract (represented by moving left to right along the bars), IFNαβ increasingly controls how viral infections develop. Bottom, right: Previous studies had shown that IFNλ is important for controlling viral infections in the intestines.
